# Variations in the Ghrelin Receptor Gene Associate with Obesity and Glucose Metabolism in Individuals with Impaired Glucose Tolerance

**DOI:** 10.1371/journal.pone.0002941

**Published:** 2008-08-13

**Authors:** Ursula Mager, Tatjana Degenhardt, Leena Pulkkinen, Marjukka Kolehmainen, Anna-Maija Tolppanen, Jaana Lindström, Johan G. Eriksson, Carsten Carlberg, Jaakko Tuomilehto, Matti Uusitupa, for the Finnish Diabetes Prevention Study Group

**Affiliations:** 1 Department of Clinical Nutrition, Food and Health Research Centre, University of Kuopio, Kuopio, Finland; 2 Department of Biosciences/Biochemistry, University of Kuopio, Kuopio, Finland; 3 Department of Health Promotion and Chronic Disease Prevention, Diabetes Unit, National Public Health Institute, Helsinki, Finland; 4 Life Science Research Unit, University of Luxembourg, Luxembourg, Luxembourg; 5 Department of General Practice and Primary Health Care, University of Helsinki, Helsinki, Finland; 6 South Ostrobothnia Central Hospital, Seinäjoki, Finland; University of Bremen, Germany

## Abstract

**Background:**

Ghrelin may influence the development of obesity through its role in the control of energy balance, food intake, and regulation of body weight. The effects of ghrelin are mediated via the growth hormone secretagogue receptor (GHSR).

**Methodology/Principal Findings:**

We genotyped 7 single nucleotide polymorphisms (SNPs) in the *GHSR* gene and assessed the association between those SNPs and obesity and type 2 diabetes-related phenotypes from 507 middle-aged overweight persons with impaired glucose tolerance participating in the Finnish Diabetes Prevention Study (DPS). Additionally, we performed *in silico* screening of the 5′-regulatory region of *GHSR* and evaluated SNPs disrupting putative transcription factor (TF) binding sites *in vitro* with gelshift assays to determine differences in protein binding between different alleles of SNPs. Rs9819506 in the promoter region of *GHSR* was associated with body weight (p = 0.036); persons with *rs9819506-AA* genotype having the lowest body weight. Individuals with *rs490683-CC* genotype displayed highest weight loss in the whole study population (p = 0.032). The false discovery rate for these results was <10%. Rs490683 and rs509035 were associated with several measures of glucose and insulin metabolism during the follow-up. Rs490683 may be a functional SNP, since gelshift experiments showed differential protein binding between the alleles, with higher binding to the G-allele. *Rs490683-C* may disrupt a putative binding site for the TF nuclear factor 1 (NF-1), thus *rs4906863-GG* genotype where the NF-1 site is intact may lead to a higher *GHSR* gene expression.

**Conclusion/Significance:**

Polymorphisms in the *GHSR* promoter may modify changes in body weight during long-term lifestyle intervention and affect ghrelin receptor signalling through modulation of *GHSR* gene expression.

## Introduction

Ghrelin is predominantly produced by the stomach and is the only known orexigenic hormone and endogenous ligand for the growth-hormone-secretagogue receptor (GHSR) [1], [2]. Ghrelin is composed of 28 amino acids and is uniquely modified by the addition of an octanoyl group to the serine residue at position three. This acylation is necessary for ghrelin to bind to the GHSR and to cross the blood-brain barrier [3].

Ghrelin is implicated in growth hormone release [2], energy balance [4], food intake, and long-term regulation of body weight [5], [6]. A major target for ghrelin is the arcuate nucleus of the hypothalamus, where ghrelin acts on appetite-stimulating groups of neurons, which express the potent orexigenic neuropeptides neuropeptide Y (NPY) and agouti-related protein (AGRP) [7]. These NPY/AGRP-containing neurons also express ghrelin receptors [8].

The *GHSR* gene, located on chromosome 3q26.31, encodes a protein that belongs to the family of G protein-coupled receptors [9]. Two distinct *GHSR* transcripts are known: GHSR type 1a acts as ghrelin receptor, whereas type 1b is a truncated form of the GHSR 1a and pharmacologically inactive [9]. GHSR 1a is expressed mainly in the hypothalamus and pituitary [10], [11], whereas the GHSR type 1b mRNA expression has also been found in many peripheral organs [10], including immune cells [12], [13], indicating that ghrelin may have multiple functions in these tissues but with thus far unknown importance.

Models of disrupted ghrelin signalling can show complex energy homeostasis-related phenotypes. *GHSR*-deficient mice show resistance to diet-induced obesity when supplied with a high-fat diet early in life, which implicates that functional ghrelin signalling is required for the full development of diet-induced obesity [1], [14].

In humans, the *GHSR* gene lies within a quantitative trait locus strongly linked to multiple phenotypes related to obesity and the metabolic syndrome [15]. Baessler *et al.* have described a “susceptible” and “non-susceptible” haplotype for obesity of five *GHSR* single nucleotide polymorphisms (SNPs) [15]. Vartiainen *et al.* [16] showed an association between insulin values and a *GHSR* variant. A recent study reports a weak association between one *GHSR* variant and BMI [17]. In recently conducted genome-wide association (GWA) studies, *GHSR* was not among the few genes associated with obesity [18], [19] or type 2 diabetes [20], [21]. Although GWA studies are very elegantly conducted to unravel the genetics of chronic diseases, candidate gene analyses also can often contribute to our knowledge about these disorders. Thus, we investigated SNPs in the *GHSR* gene and their association with obesity, glucose and insulin metabolism, and the conversion from impaired glucose tolerance (IGT) to type 2 diabetes in participants of the Finnish Diabetes Prevention Study (DPS). We demonstrated an association between one *GHSR* promoter SNP and weight loss during a 3-year follow-up and consequently showed that this SNP might have functional consequences on the expression of the *GHSR* gene.

## Results

**Figure 1:**
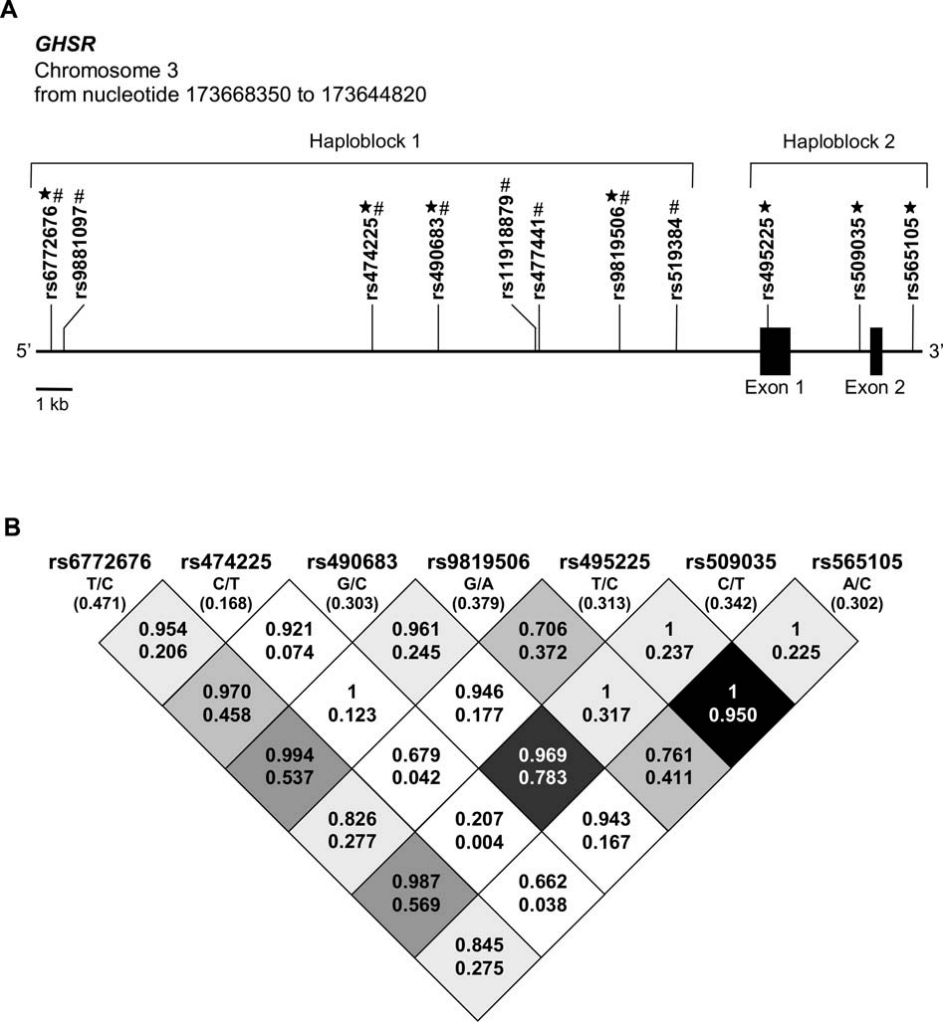
The human *GHSR* gene and location of SNPs and their LD measures. A. Schematic representation of the human *GHSR* gene indicating the locations of analyzed single nucleotide polymorphisms (SNPs) that belong to two haploblocks. (black star), SNPs genotyped in the Finnish Diabetes Prevention Study population. # SNPs from HapMap database in transcription factor (TF) binding sites and tested in gelshift assays. B. Standardized pair-wise linkage disequilibria (LD) measures of D' and r^2^ for seven *GHSR* single nucleotide polymorphisms (SNPs) of the Finnish Diabetes Prevention Study. Major/minor alleles and the minor allele frequency (in parentheses) are included below each SNP identification number. For each cell, the number on the top line is D' and the number on the bottom line is r^2^. Strength of LD is indicated in greyscale with black denoting strong and white weak LD.

We genotyped 7 SNPs in the *GHSR* gene, four of which are located in the 5′-region, one in exon 1, one intronic and one SNP in the 3′-region (Fig. 1A). Rs490683, which was associated with weight loss and measures of glucose metabolism, is in strong linkage disequilibrium (LD) with rs509035 (Fig. 1B).

### Association with obesity-related phenotypes

**Figure 2:**
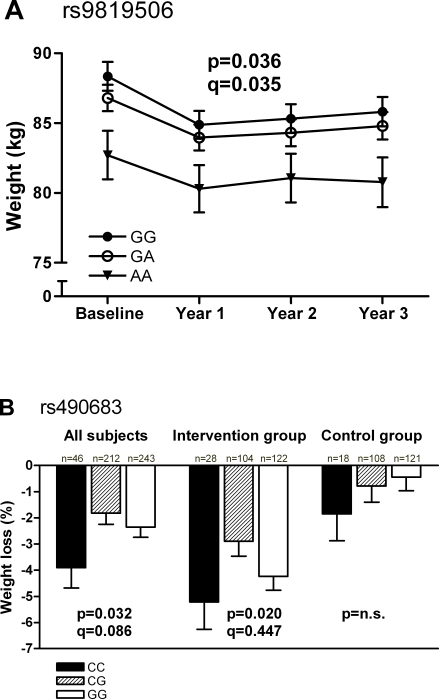
Weight and weight loss according to rs9819506 and rs490683 genotypes in DPS. A. Weight according to rs9819506 genotypes in the entire study population. Mean±SEM are estimated marginal means calculated from repeated measures ANOVA with sex and age as significant covariates. *rs9819506-GG* (n = 171, filled circles); *rs9819506-GA* (n = 200, open cirlces); *rs9819506-AA* (n = 59, filled triangles); pair-wise comparisons: *GG* vs. *GA* p = 1.000, *GG* vs. *AA* p = 0.030, *GA* vs. *AA* p = 0.116. B. Relative weight loss according to the different genotypes of rs490683 in all subjects and separately in intervention and control group. *rs490683-CC* (black bars); *rs490683-CG* (striped bars); *rs490683-GG* (white bars). The q-value denotes the false discovery rate (FDR) and is reported only for p<0.1. n.s., non-significant.

At baseline, no significant differences were observed between the genotypes of all seven SNPs for obesity-related traits (Table 1 for rs490683, data not shown for other SNPs). However, using repeated measures ANOVA we observed that during the 3-year follow-up weight was significantly different between genotypes of rs9819506 (Fig. 2A) and tended to differ for BMI (p/q = 0.056/0.041).

**Table 1:** Baseline characteristics of the DPS study subjects according to the rs490683 polymorphism of the *GHSR* gene.

	Genotype			P-value*
	*rs490683-CC* (n)	*rs490683-CG* (n)	*rs490683-GG* (n)	
Sex (M/F)	13/33 (46)	68/147 (215)	85/161 (246)	0.635^b^
Age (years)	54±7 (46)	56±7 (215)	55±7 (246)	0.246^c^
Weight (kg)	88.5±13.8 (46)	84.4±12.9 (215)	87.4±15.2 (246)	0.080^a^
BMI (kg/m^2^)	32.4±4.9 (46)	30.8±4.2 (215)	31.4±4.7 (246)	0.132^a^
Waist circumference (cm)	101.5±10.2 (46)	100.7±10.8 (213)	101.6±11.3 (246)	0.062
Waist-to-hip ratio	0.91±0.07 (46)	0.92±0.07 (213)	0.92±0.08 (246)	0.415
Fasting plasma glucose (mmol/l)	6.3±0.7 (46)	6.1±0.7 (215)	6.1±0.8 (246)	0.653
2-h plasma glucose (mmol/l)	9.0±1.6 (46)	9.0±1.6 (215)	8.8±1.4 (246)	0.198
Fasting serum insulin (pmol/l)	16.5±8.7 (40)	14.5±6.5 (193)	14.7±7.9 (228)	0.574
2-h serum insulin (pmol/l)	105.4±61.2 (39)	92.9±57.3 (192)	95.9±71.7 (227)	0.728
HOMA-IR (mmol×mU×l^−2^)	4.61±2.43 (40)	4.01±2.01 (193)	4.09±2.47 (228)	0.550
HOMA-IS (U/mol)	126.8±78.2 (40)	119.6±73.2 (193)	115.7±58.7 (228)	0.567
Total serum cholesterol (mmol/l)	5.81±0.91 (46)	5.64±0.96 (215)	5.55±0.89 (245)	0.147
HDL cholesterol (mmol/l)	1.25±0.32 (46)	1.21±0.29 (215)	1.20±0.29 (245)	0.523
LDL cholesterol (mmol/l)	3.68±0.79 (45)	3.64±0.86 (215)	3.59±0.82 (244)	0.681
Triglycerides (mmol/l)	1.95±0.92 (46)	1.74±0.76 (215)	1.68±0.75 (245)	0.139
Systolic blood pressure (mmHg)	138±17 (45)	137.4±17.8 (213)	139±18 (244)	0.458
Diastolic blood pressure (mmHg)	87±8 (45)	85±10 (213)	86±10 (244)	0.592

Values are means±SD; ANOVA for comparison among three genotype groups, adjusted for age, sex and BMI as main effects.

a p-value adjusted for age and sex.

b Chi-square Test.

c Kruskal-Wallis Test.

* FDR was >60% for all p-values.

During the 3-year intervention. all subjects being analyzed together, individuals with *rs490683-CC* genotype lost significantly more weight than others (Fig. 2B). When the two intervention groups were analyzed separately, differences in weight loss according to the genotypes were only observed in the intervention group (Fig. 2B).

No significant differences in weight loss during the intervention period were observed for other SNPs (data not shown).

We carried out haplotype analysis with the 4 genotyped SNPs in haploblock 1 (rs6772676, rs474225, rs490683 and rs9819506, respectively; Fig. 1A). The haplotype analysis did not offer more information than the single SNP analysis, thus these results were not included in the current manuscript. Concerning weight, the G-allele of rs9819506 seems to be responsible for higher weight in all subjects. The haplotype analysis regarding weight change did not reveal any significant results, largely due to the fact that not all relevant haplotypes could be tested due to their low frequency (data not shown).

### Association with phenotypes related to glucose metabolism

At baseline, no significant associations with measures of glucose metabolism and the seven genotyped *GHSR* polymorphisms were observed (Table 1 for rs490683, data not shown for other SNPs).

**Figure 3:**
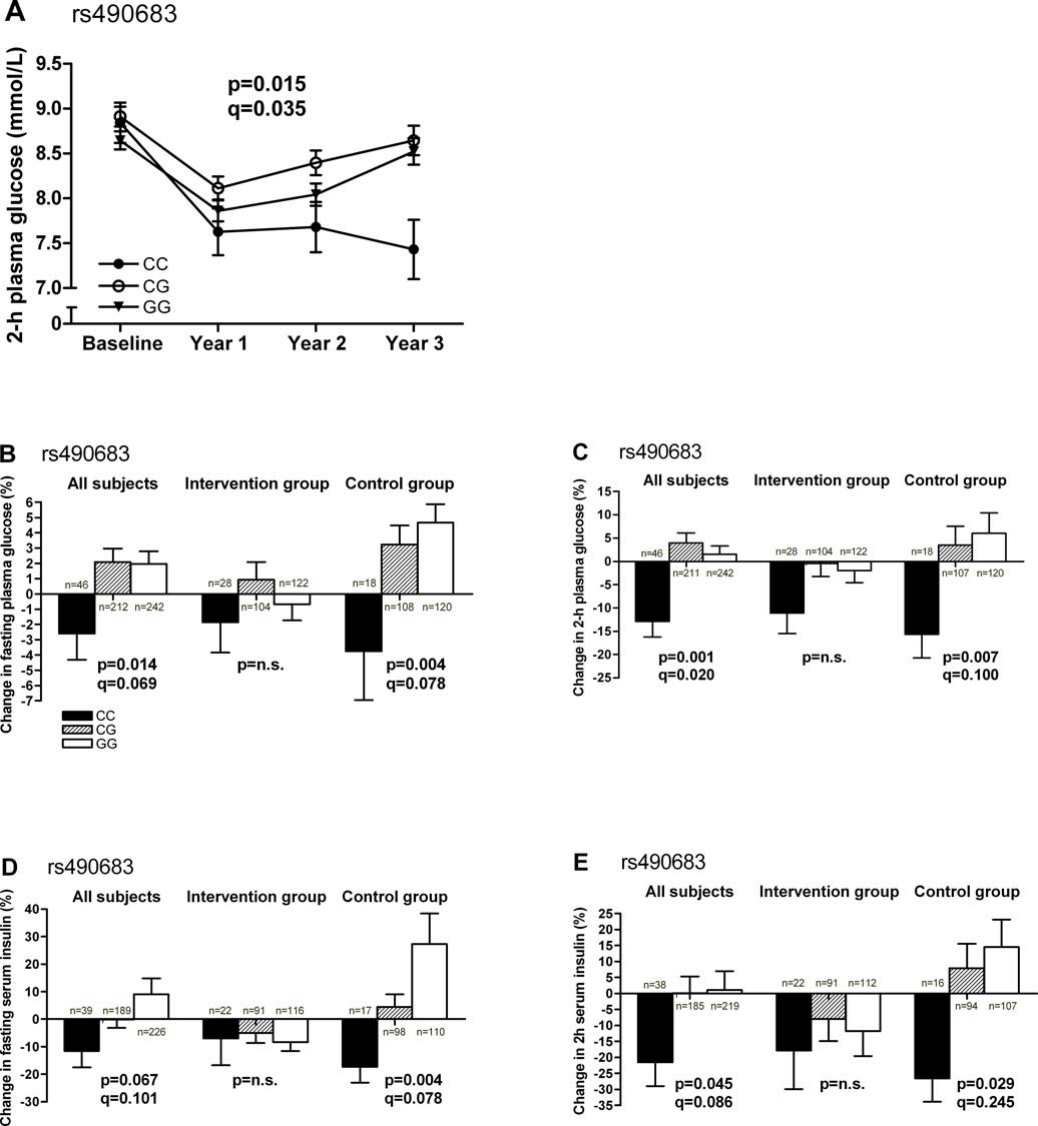
Glucose metabolism measures according to rs490683 genotypes in DPS. A. 2-h plasma glucose according to rs490683 genotypes in all subjects. Mean±SEM are estimated marginal means calculated from repeated measures ANOVA with group, age and BMI at baseline as significant covariates. *rs490683-CC* (n = 42, filled circles); *rs490683-CG* (n = 171, open circles); *rs490683-GG* (n = 210, filled triangles). Pair-wise comparisons all subjects: *CC* vs. *CG* p = 0.020, *CC* vs. *GG* p = 0.295, *CG* vs. *GG* p = 0.190. B–E. Relative changes fasting plasma glucose (B), 2-h plasma glucose (C), fasting serum insulin (D) and 2-h serum insulin (E) according to the different genotypes of rs490683 in all subjects and separately in intervention and control group. *rs490683-CC* (black bars); *rs490683-CG* (striped bars); *rs490683-GG* (white bars). The q-value denotes the false discovery rate (FDR) and is reported only for p<0.1. n.s., non-significant.

Differences between genotypes were observed with the rs6772676 SNP for fasting (p/q = 0.038/0.035) and 2-h plasma glucose (p/q = 0.006/0.035), with heterozygotes having the highest levels in the whole study population when data were analyzed longitudinally. In similar analysis for rs490683, 2-h plasma glucose (Fig. 3A) and 2-h serum insulin levels (p/q = 0.050/0.041) differed significantly according to different genotypes with *rs490683-CC* subjects tending to have the lowest 2-h glucose and insulin levels throughout the follow-up. Three-year changes in fasting plasma glucose, 2-h plasma glucose and 2-h serum insulin in the entire study population differed between rs490683 genotypes (Fig. 3B, C and E, respectively). When the study groups were analyzed separately, significant differences were found between the rs490683 genotypes in fasting and 2-h plasma glucose and fasting and 2-h serum insulin (Fig. 3B–E) in the control group, but not in the intervention group. Levels of glucose and insulin decreased in persons with *rs490683-CC* genotype during the follow-up. Furthermore, differences between genotypes were also observed with the rs509035 SNP for 2-h plasma glucose and fasting insulin in the whole study population when data were analyzed longitudinally (p/q = 0.014/0.035 and p/q = 0.037/0.035, respectively). Changes in parameters of glucose metabolism differed according to rs509035 in the whole study population (fasting plasma glucose: p/q = 0.046/0.086, 2-h plasma glucose: p/q = 0.011/0.069, 2-h serum insulin: p/q = 0.037/0.086) and in the control group separately (2-h plasma glucose: p/q = 0.038/0.267, 2-h serum insulin: p/q = 0.014/0.152), with persons with *rs509035-TT* genotype showing a decrease in those parameters during the follow-up.

None of the *GHSR* polymorphisms were statistically significantly associated with risk of type 2 diabetes (data not shown).

### Differential protein binding in the 5′-regulatory region

**Figure 4:**
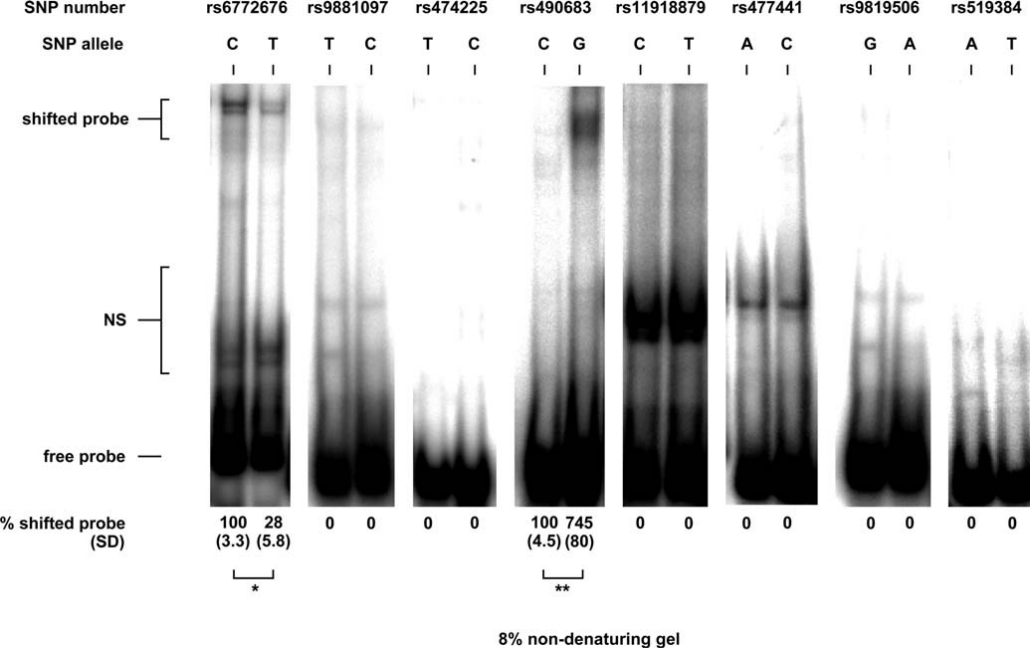
Differential protein binding of rat hypothalamus nuclear protein extract. Gelshift experiments were performed with ^32^P-labeled oligonucleotides containing one or the other allele of respective single nucleotide polymorphism (SNP). Numbers below the gels indicate the means of three independent experiments with standard deviation (SD) in parenthesis. Protein binding of the “wild-type” allele was normalized to 100%. Representative gels are shown. NS denotes non-specific DNA-protein interactions, which are not due to specific binding between the labelled DNA and the proteins contained in the hypothalamus extract. ^*^ p<0.05; ^**^ p<0.001.

We used the Genomatix MatInspector software [22] to identify putative transcription factor (TF)-binding sites in the 5′-region (haploblock 1) of the *GHSR* gene that may be implicated in the regulation of the gene expression. We screened 20 kb upstream of the translation initiation site and identified 8 SNPs that potentially disrupted one or more TF-binding sites in either one of the alleles or both. Subsequently, we tested oligonucleotides containing either of the alleles of each SNP (supplementary Table S1) to identify possible differences in protein binding between the alleles of each SNP in gelshift assays using rat hypothalami as a TF protein source. The SNPs rs6772676 and rs490683 showed different protein binding when both alleles were compared (Fig. 4). Specifically, when the protein binding of the “wild-type” allele was normalized to 100%, *rs6772676-T* allele showed lower protein binding (only 28%) compared to the *rs6772676-C* allele. Furthermore, *rs490683-G* allele showed higher protein binding (745%) compared to that of the *rs490683-C* allele. The G-allele of rs490683 is present in the core sequence of the putative binding site of the TF nuclear factor 1 (NF-1), which is disrupted when G is replaced by C (Table 2).

**Table 2:** Single nucleotide polymorphisms (SNPs) in the *GHSR* promoter and putative transcription factor (TF) binding sites.

SNP	Allele	Putative TF binding sites
rs6772676^1^^,^ 2	C	Activating transcription factor, *ATF-1*
		Pax-3 paired domain protein, expressed in embryogenesis, mutations correlate to Waardenburg Syndrome
	T	PBX - HOXA9 binding site
rs9881097	T	Avian C-type LTR TATA box
	C	Pu.1 (Pu120) Ets-like TF identified in lymphoid B-cells
		PAX 2/5/8 binding site
rs474225^1^	T	Fkh-domain factor FKHRL1 (FOXO)
		Interferon regulatory factor 3 (IRF-3)
	C	-
rs490683^1^^,^ 2	C	Orphan nuclear receptor rev-erb alpha (NR1D1), monomer binding site
	G	Non-palindromic nuclear factor I binding sites
rs11918879	C	Brn-5, POU-VI protein class (also known as emb and CNS-1)
	T	Brn-5, POU-VI protein class (also known as emb and CNS-1)
rs477441	A	Hepatic nuclear factor 1
	C	Ecotropic viral integration site 1 encoded factor, amino-terminal zinc finger domain
rs9819506^1^	G	TEA domain-containing factors, transcriptional enhancer factors 1,3,4,5
		Octamer binding site (OCT1/OCT2)
		Octamer-binding factor 1, POU-specific domain
	A	-
rs519384	A	Liver enriched Cut - Homeodomain TF HNF6 (ONECUT)
	T	Liver enriched Cut - Homeodomain TF HNF6 (ONECUT)

1 SNPs genotyped from DPS population.

2 different protein binding in gelshift assay.

## Discussion

We have shown that two SNPs (rs490683 and rs9819506) in the promoter region of the human *GHSR* gene are associated with the change in body weight and BMI. The *GHSR* SNP rs9819506, which has not been examined in previous studies, showed an association and individuals with *rs9819506-AA* genotype showed lowest values for body weight and a trend for lowest BMI during a 3-year follow-up. Interestingly, subjects with *rs490683-CC* genotype displayed highest weight loss in the whole study group as well as in the intervention group, when assessed separately. Previously, Baessler *et al.* [15] showed an association between *GHSR* haplotypes and obesity. The so-called “susceptible” haplotype tested in that study was found more often in obese individuals and included *rs490683-C* and *rs509035-T* alleles. These results might be considered contradictory to ours, where we see the *rs490683-CC* genotype as “beneficial” genotype. However, it is difficult to compare data from a longitudinal intervention study with individuals at high risk of type 2 diabetes describing the effect of the certain genotype on the weight development and a cross-sectional study carried out in a different population. A more recent study, however, could not point out a clear relationship between *GHSR* SNPs and obesity [17]. Wang *et al.* [23] showed that obese children and adolescents had a higher *rs495225-T* allele frequency than underweight subjects, but this trend could not be confirmed in their further studies. Thus, they concluded that there is no evidence for involvement of *GHSR* SNPs in body weight regulation. In our study, SNPs rs495225 and rs565105 were not unequivocally associated with measures of obesity or type 2 diabetes.

Vartiainen *et al.* [16] showed that subjects with *rs495225-CC* genotype had highest area under the insulin curve values and IGFBP-1 concentrations. In the present study, in the DPS population rs6772767, rs490683 and rs509035 were associated with several measures of glucose metabolism over four consecutive measurements. Beneficial changes in glucose metabolism could be observed specifically in individuals with *rs490683-CC* and *rs509035-TT* genotypes, especially in the control group. The control group in the DPS trial can be considered as a prospective cohort of people at high risk of diabetes. Since changes in measures of glucose metabolism during the study period were only observed in the control group, it indicates that the *rs490683-CC* genotype and the *rs509035-TT* genotype are beneficial or protective genotypes. A reduction in body weight was, however, evident when the whole study population was analysed, but this was mostly explained by the change in the intervention group. This finding is in some discrepancy with the association shown with measures of glucose metabolism. Thus, it might be that the different association with body weight and glucose metabolism are mediated via different mechanisms. In addition, we cannot exclude the impact of the moderate population size contributing to these varying associations. Unfortunately, we do not have the possibility to increase the size of the DPS population, but hopefully in the future it is possible to carry out joint analyses with the data from several other similar type of lifestyle intervention studies. However, we feel that the nature of DPS, as being a longitudinal study with an intensive lifestyle intervention in a carefully phenotyped high risk population, is unique and therefore our results offer valuable contribution and addition to the knowledge about weight and glucose control.

Rs490683 and rs509035 are in strong LD with each other and this may further explain the similar results concerning glucose metabolism phenotypes. Rs509035 is located in the intron of the *GHSR* gene, thus it was not included in our analysis for putative TF binding sites. Nevertheless, it cannot be excluded that this SNP has functional consequences, but we did not test this. Thus, based on our results we may speculate that rs490683 could be the causal SNP accounting for the effects observed.

As a limitation we need to point out that due to the distribution of these data, only non-parametric tests could be carried out where adjustments for covariates were not possible. To account for multiple testing and to control false positive rate, or type I error, we calculated the false discovery rate (FDR), expressed as q-value, for given p-values. Associations with a significant p-value, but a high q-value must be cautiously interpreted.

To test the potential functional relevance of SNPs in the 5′-region of *GHSR* we first examined the regions where SNPs are possibly disrupting putative TF-binding sites with *in silico* promoter analysis. We then tested oligonucleotides with each allele of each SNP in gelshift assays using nuclear protein extracts from rat hypothalamus. We chose to use hypothalami of male rats as a comparable protein source, due to the fact that this tissue is unavailable from a human source. The DNA sequences (oligos) used for the gelshift experiments were designed to match the human sequence. We observed that nuclear proteins were binding to the sequence containing the *rs490683-G* allele with much higher affinity than to that of *rs490683-C* allele. At this position a putative NF-1 binding site exists and is disrupted by the SNP rs490683, changing the sequence from GCCA to CCCA. The NF-1 family of site-specific DNA-binding proteins functions both as cellular TFs in the regulation of gene expression and as replication factors for adenovirus DNA replication [24]. Binding specificity of NF-1 is equivalent to both the TGGCA-binding protein [25] and the CAAT-box TF [26], [27]. NF-1 protein binds as a dimer to the dyad symmetric consensus sequence TTGGC(N_5_)GCCAA on duplex DNA, although NF-1 can also bind specifically to individual half sites (TTGGC or GCCAA) with somewhat reduced affinity [24]. NF-1 has been shown to activate transcription [24] and it can be speculated that in individuals with *rs490683-GG* genotype, where the NF-1 half site is intact, GHSR expression is enhanced, thus potentially leading to an increase in receptor signalling and ultimately to an increase in appetite [28]. In fact, our study shows that subjects with *rs490683-GG* genotype lost less weight during a lifestyle intervention than individuals with *rs490683-CC* genotype.

It has been shown recently that GHSR signals with ∼50% activity even in the absence of an agonist [29]. That implies that control of the expression level of the receptor is directly correlated to signalling activity [7]. It has been shown that during prolonged fasting, GHSR expression in the hypothalamus is increased, which could contribute to the amplification of ghrelin action [28] and could be expected to result in a ghrelin-independent increase in receptor signalling and thereby an increase in appetite [7]. The *GHSR* polymorphism rs490683 was associated with weight change in our study population and the *rs4906863-GG* genotype, where the NF-1 site is intact, might lead to increased ghrelin receptor expression.

The alleles of rs519384 did not show differential protein binding, indicating that the SNP is not in an important region for GHSR gene expression. Although rs9819506 was associated with weight and BMI, gelshift assays showed no difference in protein binding between the alleles, which may be due to species differences. Although we could observe a difference in protein binding between the alleles of rs6772676, it cannot explain the observed association with glucose levels.

TFs and more importantly their DNA binding domains are highly conserved between humans and rodents. For example, the nucleic acid sequence of the rat NF-1 is 99% identical to the human sequence and on amino acid level there is a 91% overlap between the sequences. The variations in the rat NF-1 protein (compared to the human sequence) are not located within its DNA binding domain, thus they are unlikely to affect its binding to DNA. Due to the tissue unavailability from humans the rat protein extract provides a good model to test for differential protein binding to the alleles because of the high degree of conservation between the two species. We used hypothalami of male rats only, but obviously the autosomes of male and female rats are identical. Only genes located on the Y-chromosome will differ between the two sexes, whereas e.g. NF-1 is located on chromosome 17. Some TFs might show a difference in their expression levels. Thus, the gelshift bands might be stronger or weaker comparing males and females but the clear difference of protein binding to the different SNP alleles will still occur.

To our knowledge, no GWA study for obesity has been carried out in Finns alone; however, samples from Finnish studies have been used in replication studies (e.g. [18], [19]). The primary GWA studies investigating the genetics of type 2 diabetes are available. The studies regarding type 2 diabetes in Finnish subjects [20], [21], [30] have used the Illumina HumanHap300 Genotyping BeadChips containing over 317,000 SNPs. Two SNPs in the *GHSR* gene overlap with the SNPs investigated in our study, that is rs9819506 and rs565105. One more SNP, rs10513702, is in high LD (r^2^ = 0.966) with rs6772676, which was also genotyped in our study. Other GWA studies investigating obesity have used variably the Affymetrix GeneChip Human Mapping 10K, 100K or 500K Array Set [31], [32], [33], [34]. In the 500K Array Set are 6 SNPs of the *GHSR* gene included and one of those overlaps with the SNPs genotyped in our study (rs509035). The other *GHSR* SNPs are not in LD with SNPs which we genotyped. In GWA studies stringent quality control criteria are applied for the given setting. Thus it is possible that the few SNPs used in this study from the *GHSR* gene did not match those criteria (e.g. call rate, Hardy-Weinberg equilibrium for the given study population).

In summary, results of our study suggest that polymorphisms in the *GHSR* gene are longitudinally associated with obesity and glucose metabolism in a prospective study setting. Genetic variation in the promoter may have an effect on appetite regulation by modulating gene expression of GHSR and subsequently ghrelin receptor signalling. This study is the first to show potential functional consequences of a *GHSR* SNP. Further studies are warranted to confirm our results and to find SNPs that are causal and have clinical relevance.

## Materials and Methods

### Subjects and study design

The Finnish DPS is an intervention study carried out in five centres in Finland (clinical trial reg. no: NCT00518167). The main aim of the study was to assess the efficacy of an intensive individually designed diet and exercise program to prevent or delay the onset of type 2 diabetes in subjects with IGT. The study design, subjects, inclusion and exclusion criteria and intervention program have been described earlier in detail [35], [36]. In brief, 522 overweight (BMI≥25 kg/m^2^) men and women aged 40 to 65 years with IGT participated in the study. DNA samples were available from 507 individuals (intervention group 259 and control group 248 subjects). IGT was defined as a 2-h plasma glucose concentration of 7.8 to 11.0 mmol/l (oral glucose tolerance test [OGTT] 75 g) with a fasting plasma glucose concentration less than 7.8 mmol/l [37]. The persons enrolled in the study were randomly assigned to one of the two treatment modalities, the intervention group or the conventional care control group. The study protocol was approved by the Ethics Committee of the National Public Health Institute in Helsinki, Finland, and conducted in accordance with the guidelines proposed in the Declaration of Helsinki. All participants gave written informed consent. We certify that all applicable institutional and governmental regulations concerning the ethical use of human volunteers were followed during this research.

### Selection of *GHSR* SNPs and genotype analysis

For selection of *GHSR* SNPs for genotype analysis the HapMap [38], NCBI [39] and Ensembl [40] databases were used. Originally, six tag SNPs from two haploblocks covering 15 kb of the gene including three SNPs in haploblock 1 in the 5′ end of the gene (rs474225, rs490683 (alternative name in HapMap rs863441), rs9819506), and three SNPs in haploblock 2, one in the coding region in exon 1 leading to a synonymous amino acid substitution (rs495225), one in the intron (rs509035) and one in the 3′ end of the *GHSR* gene (rs565105) were selected (Fig. 1A). In addition, all SNPs in haploblock 1 in the 5′ regulatory region (spanning over 17 kb, chr 3: 173648897–17365897; NCBI accession number for *GHSR* NM_198407.1) available in the HapMap database and located in putative TF-binding sites were tested for their functionality in gelshift experiments. SNPs which showed different protein binding *in vitro* were genotyped additionally (rs6772676).

Genotyping was performed with TaqMan Allelic Discrimination Assays (Applied Biosystems, Foster City, CA, USA). The PCR amplification was performed in a GeneAmp PCR system 2700 (Applied Biosystems, Foster City, CA, USA) in following conditions: 95°C for 10 min and 40 cycles of denaturation 92°C for 15 sec and annealing/extension 60°C for 1 min. Allele-specific fluorescence was detected on an ABI Prism 7000 sequence detector. A subset of randomly selected samples representing 6.3% of the study cohort was repeated.

### Promoter analysis

*In silico* promoter analysis of the sequences containing all SNPs in haploblock 1 for putative TF-binding sites was performed using Genomatix MatInspector Release professional 7.4.8.2 [22]. We determined all SNPs that lie in possible TF-binding sites and consequently all TF-binding sites that are disrupted by either one of each SNP allele. We used a threshold of 0.75 for core similarity and the optimized setting for matrix similarity.

### Protein extraction

Hypothalami of three male Sprague-Dawley rats (supplier Harlan, Netherlands) were pooled and the frozen tissue was homogenized in 0.5 ml of low salt buffer (10 mM Hepes, pH 7.9, 1.5 mM MgCl_2_, 50 mM KCl, 0.5 mM dithiothreitol, proteinase inhibitors) using a rotor-stator homogenizer (ART Labortechnik, Müllheim, Germany). Cells were then resuspended in 2–3 packed volumes of the same low salt buffer with 0.5% Nonidet P-40 and homogenized by pipetting on ice. The nuclear pellet was subsequently resuspended in one packed cell volume of high salt buffer (10 mM Hepes, pH 7.9, 25% glycerol, 420 mM NaCl, 1.5 mM MgCl_2_, 0.2 mM EDTA, 0.5 mM dithiothreitol, proteinase inhibitors). Extraction was performed for 30 min on ice.

### Gelshift assay

Gelshift assays were performed with 10 µg protein of the nuclear extracts. The proteins were incubated for 15 min in a total volume of 20 µl of binding buffer (150 mM KCl, 1 mM dithiothreitol, 25 ng/ml herring sperm DNA, 5% glycerol, 10 mM Hepes, pH 7.9). Constant amounts (1 ng) of ^32^P-labeled double-stranded oligonucleotides (50,000 cpm) containing either one of the respective SNP alleles (supplementary Table S1) were then added, and incubation was continued for 20 min at room temperature. Protein-DNA complexes were resolved by electrophoresis in 8% non-denaturing polyacrylamide gels (mono- to bisacrylamide ratio 19∶1) in 0.5×TBE (45 mM Tris, 45 mM boric acid, 1 mM EDTA, pH 8.3) for 90 min at 200 V and quantified on a FLA-3000 reader (Fuji, Tokyo, Japan) using ScienceLab99 software (Fuji).

### Statistical analysis

The significances of differences in genotype and allele frequencies were analyzed using a chi-square test and a two-sided Fisher's exact test, respectively. Normal distributions were tested with the Kolmogorov-Smirnov test with Lilliefors correction. To normalize the skewed distributions, logarithmic transformations or reciprocal transformations were applied when needed. The differences in continuous variables between genotypes of single SNPs at baseline were evaluated with the general linear model (GLM) for the univariate analysis of variance (ANOVA). Biochemical measurements were adjusted for age, sex and BMI as main effects and weight-related variables for age and sex only. When variables could not be transformed to be normally distributed, Kruskal-Wallis test was used. GLM repeated measures ANOVA was used to analyze differences between genotypes of each SNP during the follow-up period concerning obesity-related and insulin- and glucose-metabolism related phenotypes. A final model was built manually with a backward selection procedure with following covariates included: age at baseline, sex, study group and BMI at baseline, as appropriate. Genotype*group interaction was tested and groups were analyzed separately if the interaction term was statistically significant. P-values are for trend between all three genotype groups and pairwise comparisons based on estimated marginal means were adjusted for multiple comparisons with Bonferroni correction. Relative changes in weight and measures of glucose and insulin metabolism from baseline to year 3 were analyzed with Kruskal-Wallis test, because data were not normally distributed even after applying several transformations. A p-value of 0.05 or lower was considered statistically significant. Correction for multiple hypothesis testing was performed with FDR using the Q-value 1.0 software [41]. π_0_ was estimated with bootstrap method using a λ range from 0 to 0.9 by 0.05. The FDR for each p-value is reported as q-value. The q-value threshold is the proportion of significant features that turn out to be false leads. Data are given as means±SD, unless otherwise stated. All data were analyzed using SPSS for Windows 14.0 (SPSS Inc., Chicago, IL, USA). LD statistics were calculated and haplotype blocks were visualized by using the Haploview software [42].

## Supporting Information

Table S1 Oligonucleotides used in gelshift assays. List of oligonucleotides which were used to test the difference in protein binding among different GHSR SNP alleles.
(0.04 MB DOC)
