# Supplementary material for: Variations in the Ghrelin Receptor Gene Associate with Obesity and Glucose Metabolism in Individuals with Impaired Glucose Tolerance
Source: PLoS One. 2008 Aug 13;3(8):e2941. doi: 10.1371/journal.pone.0002941 (PMC2491902; doi:10.1371/journal.pone.0002941)
Supplement: Table S1 — Oligonucleotides used in gelshift assays. List of oligonucleotides which were used to test the difference in protein binding among different GHSR SNP alleles. (0.04 MB DOC) [file pone.0002941.s001.doc]

**Supplemental Table S1.** Oligonucleotides used in gelshift assays.

| **Polymorphism** | **Oligo sequence 5’-3’** |
| --- | --- |
| rs6772676 | agcctcaacacctga**c**gatttttcaggg |
|  | agcctcaacacctga**t**gatttttcaggg |
| rs9881097 | ggttctaccttcct**t**agttaagcttcatcc |
|  | ggttctaccttcct**c**agttaagcttcatcc |
| rs474225 | gcctttg**t**ttccctttcatct |
|  | gcctttg**c**ttccctttcatct |
| rs490683 | ccatataaaagaggt**c**ccagaaagct |
|  | ccatataaaagaggt**g**ccagaaagct |
| rs11918879 | actttttaataaatgatg**c**tgaaacaactg |
|  | actttttaataaatgatg**t**tgaaacaactg |
| rs477441 | actaaagcca**a**aaaataatagaaca |
|  | actaaagcca**c**aaaataatagaaca |
| rs9819506 | agctggaat**g**catataagtgacat |
|  | agctggaat**a**catataagtgacat |
| rs519384 | ttaaaaactc**a**atttggggccg |
|  | ttaaaaactc**t**atttggggccg |
